# Optimism persists when walking in unpredictable environments

**DOI:** 10.1038/s41598-023-33662-6

**Published:** 2023-04-26

**Authors:** Mary A. Bucklin, Jasjit Deol, Geoffrey Brown, Eric J. Perreault, Keith E. Gordon

**Affiliations:** 1 Department of Physical Therapy and Human Movement Sciences, Feinberg School of Medicine, Northwestern University, 645 N. Michigan Ave, Suite 1100, Chicago, IL 60611 USA; 2 Department of Biomedical Engineering, Northwestern University, Evanston, IL USA; 3 Shirley Ryan Ability Lab, Chicago, IL USA; 4 Department of Physical Medicine and Rehabilitation, Northwestern University, Chicago, IL USA; 5 Research Service, Edward Hines Jr. VA Hospital, Hines, IL USA

**Keywords:** Motor control, Learning and memory

## Abstract

Humans continuously modulate their control strategies during walking based on their ability to anticipate disturbances. However, how people adapt and use motor plans to create stable walking in unpredictable environments is not well understood. Our purpose was to investigate how people adapt motor plans when walking in a novel and unpredictable environment. We evaluated the whole-body center of mass (COM) trajectory of participants as they performed repetitions of a discrete goal-directed walking task during which a laterally-directed force field was applied to the COM. The force field was proportional in magnitude to forward walking velocity and randomly directed towards either the right or left each trial. We hypothesized that people would adapt a control strategy to reduce the COM lateral deviations created by the unpredictable force field. In support of our hypothesis, we found that with practice the magnitude of COM lateral deviation was reduced by 28% (force field left) and 44% (force field right). Participants adapted two distinct unilateral strategies, implemented regardless of if the force field was applied to the right or to the left, that collectively created a bilateral resistance to the unpredictable force field. These strategies included an anticipatory postural adjustment to resist against forces applied to the left, and a more lateral first step to resist against forces applied to the right. In addition, during catch trials when the force field was unexpectedly removed, participants exhibited trajectories similar to baseline trials. These findings were consistent with an impedance control strategy that provides a robust resistance to unpredictable perturbations. However, we also found evidence that participants made predictive adaptations in response to their immediate experience that persisted for three trials. Due to the unpredictable nature of the force field, this predictive strategy would sometimes result in greater lateral deviations when the prediction was incorrect. The presence of these competing control strategies may have long term benefits by allowing the nervous system to identify the best overall control strategy to use in a novel environment.

## Introduction

In novel environments humans continuously modulate their gait based on their experience to plan for both anticipated and unpredictable disturbances. The control strategies^1–4^ that we implement depends on our ability to anticipate these disturbances. For example, when walking a strong dog on a leash, based on our prior experience with the animal, we will prepare to resist the movement of the dog as soon as it fixates on an approaching cat. In this situation, a feedforward predictive model^5–7^ allows for efficient generation of forces that specifically oppose the pulling dog and maintain our desired walking path. However, our plan should not be so specific if the dog is walked on a crowded street with many attractions. Although it is likely that the animal will regularly pull on the leash, the direction of those pulls may change quickly and unpredictably as the dog shifts its attention from one attraction to the next. In this situation, an alternative strategy could be to co-contract muscles in the body^8,9^ or change body postures^10–13^, to be more resistant to the dog’s movements regardless of direction. In the field of motor control, strategies that increase the body’s overall resistance to external displacements, are known as impedance control^12,14^. Prioritization of different control strategies (predictive or impedance control) will likely have consequential trade-offs during walking (stability^15^, energetic efficiency^16,17^, maneuverability^18^ etc.). However, how people adapt these two forms of motor planning to create stable walking in uncertain environments is not well understood. Thus, this study sought to understand how people adapt their motor plans as they repetitively move through an unpredictable external force field by observing changes in their walking trajectories.

During forward walking our whole body center of mass (COM) naturally oscillates in multiple directions relative to our base of support^19^. In particular, the requirements of the nervous system to select strategies to successfully control medio-lateral motions of the whole body center of mass (COM) are considerable^20–22^. Beginning at toe-off, the lateral velocity of the COM is relatively large and directed towards the stance limb. To maintain a straight-ahead walking trajectory, this lateral velocity must be reduced to zero (occurring around midstance), and then redirected towards the midline. Failure to arrest the lateral momentum of the COM will result in motion beyond the lateral base of support border (determined by the stance limb medio-lateral foot-placement). COM travel beyond the lateral base of support border will require a corrective step(s) to prevent a fall and restore the desired forward walking trajectory. The lateral motion of the center of mass is also approximately periodic. Appropriately timed muscle actions anticipate this periodic movement and are used for efficient control. When the external environment produces changes in this motion in a predictable manner, our nervous system can use the difference between anticipated and actual motor errors to update our predictive control system^23^.

If the environment is frequently changing, research suggests that error sensitivity is reduced, meaning that people will suppress updates to their predictive control system in response to movement errors because a compensatory response may increase rather than decrease error on future movements^24,25^. To deal with uncertainty, the nervous system may instead adapt an impedance control strategy. Impedance describes the dynamic relationship between imposed motions and the forces generated in response^12,14^. Impedance can be regulated through changes in muscle co-contraction^26^, posture^10,27^, or spinal and supraspinal feedback gains^28,29^. An advantage of an impedance control strategy is that if small movement errors or perturbations occur, the body can recover from these disruptions with minimal time delays. There is evidence that in certain situations people will adapt gait patterns (i.e. short, wide, and fast steps) to provide a robust resistance to pseudorandom external perturbations^30–33^. These kinematic changes would increase the impedance of the trunk in the frontal plane, providing increased resistance to unexpected disturbances from either side. In contrast, others have found no changes in gait kinematics when walking in unpredictable environments^34–37^, suggesting that strategies to increase whole body impedance through stepping pattern and body posture modulations were not employed. These differences in kinematic strategies between studies are difficult to evaluate without first understanding the underlying motor planning strategy.

In the current experiment, to better understand how people adapt to create stable walking trajectories in unpredictable environments, we assessed a series of discrete walking trials performed in an unpredictable environment that applied destabilizing external forces directly to the body. The iterative experimental design allowed us to evaluate trial-by-trial adaptations while the application and removal of external forces will be used to investigate underlying control mechanisms^8,9,14^. We previously found this experimental design valuable for understanding how people plan and control walking in novel and predictable environments^38,39^. In the current study, the following series of events, as were seen in the previous studies, suggests a predictive control strategy. When participants initially interact with a predictable force field, they experience a COM trajectory deviation in the direction of the force field. With practice they generate body forces specific to oppose the force field, thereby adapting their COM trajectory back towards baseline value. Finally, when the force field is unexpectedly removed, the learned compensatory forces result in and ‘after effect’ causing the COM to move opposite to the direction of their initial error. Similarly, in the current study, the following series of events would be evidence of an impedance control strategy. When participants initially interact with a force field—whether predictable or not—they experience COM trajectory deviation in the direction of the force field. With practice they adopt kinematic profiles, increase muscle co-contraction, or alter feedback pathways to increase the impedance of the COM to reduce deviations in response to perturbations from any direction. This strategy would also adapt their COM trajectory back towards baseline value, but when the force field is unexpectedly removed, there would be no after effect because compensation via impedance control does not involve the generation of net forces as can be used when disturbances to the COM motion are predictable.

There are a variety of strategies that could be implemented to control impedance during walking. These include controlling whole-body stiffness via co-activation of antagonistic muscle pairs^8,9,14^, exploitation of posture^10–13^ , and tuning of reflex gains^29,40^. In our previous research^38^ ,we observed two postural control strategies that were particularly valuable for resisting strong laterally-directed external forces during goal directed walking (walking from a start to an end target). These strategies included modulating lateral foot placement of the stepping limb during walking and increasing the lateral translation of the COM during the anticipatory postural adjustment occurring during gait initiation. Exploitation of posture at the trunk^41^ and lower limb^42–45^ are both well recognized strategies to control lateral motion of the COM during walking^20,46^. However, both these strategies are only effective for resisting COM movements in a single direction. If a person takes a wide step to the right, their body will be ideally positioned to resist external forces that accelerate the COM to the right but poorly positioned to resist external forces that accelerate the COM to the left. Similarly, increasing the lateral translation of the COM towards the left during gait initiation will position the body to resist forces applied to the right but not the left when forward walking begins. Thus, it is not clear if or how the nervous system may use these directionally specific strategies when walking in an unpredictable environment that has the potential to create perturbations in either the right or left direction.

Our purpose was to investigate how people adapt both predictive and impedance control strategies when walking in an unpredictable environment. To do this, we evaluated COM trajectories during a goal-directed walking task in a novel and unpredictable environment. The environment was created by applying continuous, laterally-directed force fields to the COM that were proportional in magnitude to forward walking velocity and randomly alternated between being directed to the right and left each walking trial. We hypothesized that when performing a goal-directed walking task in a novel and unpredictable environment, people would adapt a control strategy that would reduce the magnitude of the COM lateral deviations created by the unpredictable force field. We performed two analyses to assess if the mechanism for reducing the COM lateral deviations was associated with predictive or impedance control strategies. First, we examined COM trajectories during catch trials when the force field was unexpectedly removed. Catch trial trajectories resembling trajectories during baseline trials would suggest the implementation of an impedance control strategy in the unpredictable force field, while trajectory errors in the opposite direction of the proceeding force field trial would be suggestive of a predictive control strategy. Second, we evaluated trial-by-trial adaptations to determine how COM lateral deviations were influenced by the force field direction on prior trials. Outcomes from this analysis would indicate if trial-by-trial adaptations were either impedance control or predictive. Additionally, we evaluated foot placement (lateral foot placement) and anticipatory postural adjustments (COM lateral offset) to evaluate the strategies used to create the motor control mechanisms.

## Methods

### Participants

Fifteen healthy young adults (8 females, 25.5 ± 3.6 years and 63.3 ± 9.9 kg, mean ± SD) participated. The Northwestern University Institutional Review Board approved the protocol and all participants provided informed written consent. All methods were performed in accordance with relevant guidelines and regulations. Participant included being able to walk continuously for 30 min without fatigue or health risk and that they were free of any musculoskeletal and/or vestibular pathologies affecting gait or balance.

### Experimental setup

**Figure 1:**
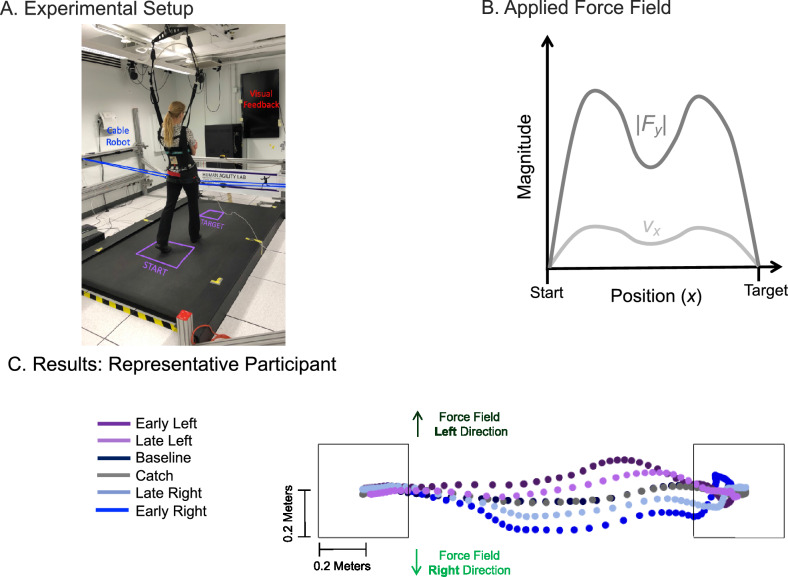
Experiment overview (**A**) Experimental setup, (**B**) Applied force field, and (**C**) Results: representative participant (**A**) Schematic top view of a participant shown performing the goal-directed walking task, walking from the start to the end target. The cable robot consisted of a pair of actuated cables (dashed black lines), routed through a trolley system (black circles), and attached bilaterally (grey circles) on the medial aspect of a snug pelvic harness worn by the participant. Force on each cable was controlled by a series-elastic linear motor (grey boxes). *F*_*y*_* (*dark grey vector) represents force applied to the COM and *v*_*x*_ (light grey vector) represents forward walking velocity_*.*_ (**B**) Representation of the force applied to the participant’s COM during a force field trial. Force applied (dark grey trace) is proportional in magnitude to forward walking velocity (light grey trace) with a gain of 80 N/(m/s) and was applied in a randomized order towards either towards the participant’s right or left side each trial. (**C**) COM trajectory during select trials from analysis periods for a single representative participant.

Participants preformed a series of discrete goal-directed stepping trials, stepping from a start target to an end target (Fig. 1a). The targets (0.4 × 0.4 m square) were projected (Hitachi America, Ltd) on the walking surface. The distance between targets was adjusted for each participant to be 1.5 × leg length, approximately a two-step task. For safety, participants wore a trunk harness attached to a passive overhead safety device (Aretech, Ashburn, VA) that moved freely on an overhead trolley in the fore-aft direction. The harness shoulder straps were adjusted so that they did not provide bodyweight support or restrict lateral movements during the trials.

To create an unpredictable environment, a cable robot applied a laterally-directed force field to the COM during walking (Fig. 1a)^47^. The force field was proportional in magnitude to forward walking velocity and applied in a random order towards either the participant’s right or left side (Fig. 1b). Forward walking velocity was calculated in real-time using the derivative of COM position measured by a string potentiometer. Position data was sampled at 100 Hz, and low pass filtered to reject spikes in the derivative (velocity) due to noise. Prior to starting each trial, participants were not informed and could not detect if the force field would be applied or in what direction it would be applied. To ensure that participants began forward walking before experiencing the force field, a forward velocity threshold of 0.26 m/s was used to trigger the force field onset. The applied force field gain (80 N/(m/s)) was the same across participants and selected to provide a challenging walking environment, but not strong enough to evoke a fall.

To ensure similar forward walking velocities across all trials, participants received feedback at the end of each trial and were instructed to modify their next trial accordingly. A monitor positioned at the end of the walking path provided visual feedback stating either “too slow”, “too fast”, or “success”, depending on how the peak forward velocity compared to a desired value of 1.2 ± 0.1 m/s. The goal of this feedback was to encourage participants to maintain a consistent speed such that they would experience an approximately consistent force field each trial. Therefore, all trials were included in analysis, even if they were “too slow” or “too fast”.

We placed 13 active markers on the pelvis and bilaterally on the greater trochanter, lateral malleolus, calcaneus, and 2nd and 5th metatarsals to measure kinematics. A 12-camera motion capture system (Qualisys, Gothenburg Sweden) recorded 3D marker coordinates at 200 Hz.

### Protocol

**Figure 2:**
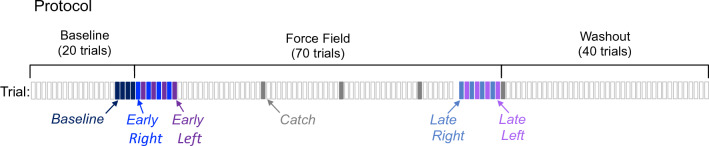
Protocol Participants performed 130 consecutive trials, each represented as a rectangular block ordered left to right. The force field was applied during Force Field trials (except for catch trials) and absent during Baseline and Washout trials. Data was analyzed during six distinct experimental four-trial periods highlighted in their respective colors.

Participants performed 130 consecutive goal-directed walking trials (Fig. 2), consisting of 20 Baseline trials (no applied forces), followed by 70 Force Field trials (forces applied randomly to either the right or left), and concluding with 40 Washout trials (no applied forces). Additionally, three catch trials (no applied forces) were interspersed within the Force Field trials occurring during trial numbers 45, 60, and 75. Catch trials were used to evaluate control strategies. Participants were not aware of trial order.

Before starting the stepping task, participants first practiced the task without any forces applied to become familiar with the target speed requirement. Then participants performed two trials in the force field (one right directed field and one left) when they were explicitly told the direction of the field before each trial. This experience was provided for safety to ensure participants had a general awareness of they may experience during the upcoming experiment. Besides these preliminary trials, participants did not have any prior experience to walking in this robotic device.”

Each stepping trial began with the participant standing stationary with both feet located in the start target. Next, participants heard an auditory “get ready” command followed by a “beep” cue. At the cue, participants walked quickly to the end target, stepping with their right foot first. The trial concluded when both feet were located within the end target. At this time, a second auditory “beep” signaled the trial was over and that the participant should return to the start target. No forces were applied to the participant as they reset their position to the start target. Participants could take as many steps as needed to accomplish the task. To minimize upper limb movements, participants crossed their arms during the trials. Besides these provisions, participants were told to complete the task in the manner they felt most comfortable.

### Data processing and calculations.

Kinematic marker data was processed using Visual3D (C-Motion, Germantown, MD) and a custom MATLAB (MathWorks, Natick, MA) program. Marker data was gap-filled and low-pass filtered (Butterworth, 6 Hz cut-off frequency). Gait events, time of initial foot contact and toe-off, were identified by the inferior-superior positions of the calcaneus and 2nd metatarsal markers for each step. Initial contact was identified as the local minimum of the calcaneal marker per step and toe-off as the local minimum of the 2nd metatarsal per step. All steps were visually inspected to verify accurate event detection. COM position was calculated in Visual3D as the center of the pelvis model, determined by three pelvic and two greater trochanter markers.

**Figure 3:**
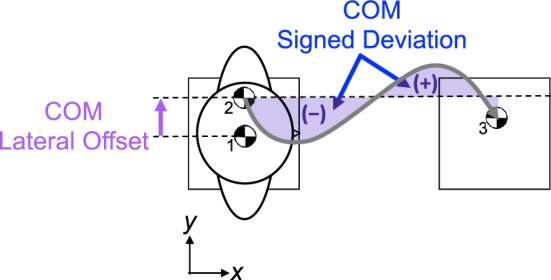
Center of Mass Data Analysis COM lateral offset, shown in purple, was defined as the lateral distance between the COM location before the “GO” cue (1) and COM location at first toe-off (2). This value represents lateral excursion of the COM prior to forward movement. The grey trajectory represents the COM motion during the trial. COM signed deviation was defined as the blue shaded area between the grey trajectory and a straight-line path originating from (2) and ending at (3). Deviations to the right of the straight-line path were given negative values, and deviations to the left were positive. Positive and negative deviations were added together for the total signed deviation.

To measure control of COM trajectory, we analyzed kinematic data of COM trajectory between start and end targets. To evaluate control of COM trajectory we calculated a normalized COM signed deviation (COM signed deviation), the signed area of COM trajectory relative to a straight-line path originating from the lateral COM position at first toe-off, minus the average of COM signed deviation throughout Baseline (Fig. 3). COM signed deviation reflects directional biases in COM trajectory relative to baseline performance.

To gain insight into the strategies that participants used to create their COM trajectory we evaluated anticipatory postural adjustments (APA’s)^48,49^ by quantifying the lateral movement (kinematics) of the COM prior to forward movement. We calculated this COM lateral offset as the lateral distance between the COM position at the “GO” cue, and the COM position at first toe-off (Fig. 3). Additionally, we evaluated lateral foot placement, calculated as the medio-lateral distance between the COM and 5th metatarsal markers at initial foot contact. We evaluated lateral foot placement for the first two steps of each trial.

### Statistical Analysis

We hypothesized that when performing a goal-directed walking task in a novel and unpredictable environment, people would adapt a control strategy to reduce the magnitude of the COM lateral deviations created by the unpredictable force field. We tested this hypothesis by evaluating if the magnitude of the COM Signed Deviation changed from the initial to final trials in the force field. We performed two analyses to gain insight into the control strategy people used. First, we examined COM trajectories during catch trials when the force field was unexpectedly removed. If participants were using an impedance control strategy, we would anticipate that COM trajectories would be similar during baseline and catch trials. Second, we evaluated trial-by-trials adaptations to assess how the force field direction on a given trial influenced participants’ response COM signed deviation on the current and future trials. Finally, to understand the mechanisms participants used to interacted with the force field we examined if anticipatory postural adjustments (COM lateral offset) and foot placement (lateral foot placement) were adapted in the force field.

For this statistical analysis, we averaged four trials at seven different experimental periods defined within the 130 consecutive stepping trials (Fig. 2).

1. *Baseline* performance was estimated as an average of trials 17–20 (last four trials of Baseline).
2. *Early field right* was an average of the first four trials when the force field was applied to the right (first four right trials in the Force Field).
3. *Early field left* was an average of the first four trials when the force field was applied to the left (first four left trials in the Force Field).
4. *Late field right *was an average of the last four trials when the force field was applied to the right (last four right trials in the Force Field).
5. *Late field left* was an average of the last four trials when the force field was applied to the left (last four left trials in the Force Field).
6. *Catch right* was an average of trials 45, 60, 75, and 90 (three catch trials and first trial of Washout) for trials that were immediately preceded by a force field right trial.
7. *Catch left* was an average of trials 45, 60, 75, and 90 (three catch trials and first trial of Washout) for trials that were immediately preceded by a force field left trial.

For COM signed deviation and lateral foot placement, each individual trial was normalized to baseline prior to averaging for experimental periods. For each participant we calculated these average performance estimates for four experimental periods (early field right, early field left, late field right, and late field left) for each metric (COM signed deviation, COM lateral offset, and lateral foot placement) and calculated three additional experimental periods for COM signed deviation (baseline, catch right, and catch left) Due to directionality differences, for COM signed deviation, we took the absolute value of early field right, early field left, late field right, and late field left, prior to statistical analyses.

The following statistical analyses were conducted using SPSS (IBM, Armonk, NY). We performed two-by-two repeated measures ANOVAs with a within subject factor of direction (right or left) and experimental period (early or late) to evaluate COM signed deviation, COM lateral offset, and lateral foot placement. Additionally, we performed one-way repeated measures ANOVA with a within subject factor of experimental period (baseline, catch right, and catch left) to evaluate COM signed deviation. When a significant main effect was found, Bonferroni-corrected pairwise comparisons were made between experimental periods. Three pairwise comparisons were made (baseline—catch right, baseline—catch left, and catch right—catch left) to evaluate COM signed deviation. Significance was set at the *p* < 0.05 level for the ANOVAs and pairwise comparisons.

To further test our hypothesis that COM signed deviation (COM_err_) was controlled using an impedance control strategy rather than a predictive control strategy, we evaluated trial-by-trials adaptations. Specifically, we used a finite impulse response function to estimate how the COM signed deviation (COM_err_) on a given trial was influenced by the force field directions (d) in the current trial and on the five trials immediately preceding it (Eq. 1). Parameters (a_i_) of the finite impulse response function were estimated using a linear regression. The analysis was conducted using MATLAB (MathWorks, Natick, MA). Significance was set a *p* < 0.05 level for all comparisons.

1 $$COM_{{err_{j} }} \; = {\text{ }}\;\sum\limits_{{i = 0}}^{5} {a_{i} d_{{j - i}} } {\text{ }}$$

## Results

### Center-of-mass signed deviation—early to late

**Figure 4:**
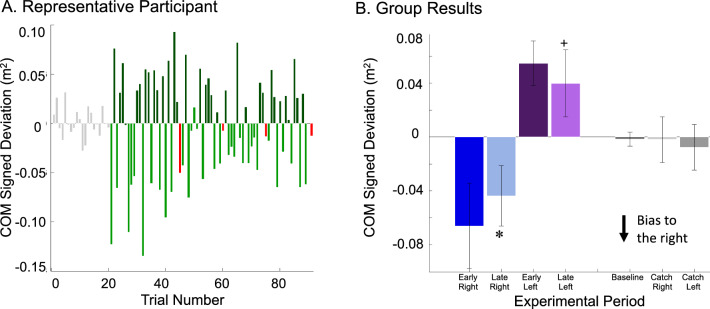
COM signed deviation (**A**) Representative participant and (**B**) Statistical results (**A**) Representative data for a single participant’s COM signed deviation are shown across experimental trial. Grey boxes represent Baseline periods when the force field was off. Light green represents trials when the force field was applied to the right. Dark green represents trials when the force field was applied to the left. Red represents catch trials when the force field was off. (**B**) Mean $\pm$ SD for COM signed deviation across experimental period. X-axis labels: ER, EL, LR, LL, B, RC, and LC represent periods early right, early left, late right, late left, baseline, right catch, and left catch, respectively. Significance (*p* < 0.05) from early field right is denoted by (✽) and significance (*p* < 0.05) from early field left is denoted by ( +). Outcomes from the two-by-two ANOVA, which analyzed adaptation, identified a significant main effect of experimental period (*p* = 0.001) indicating that the magnitude of the COM signed deviation was greater during the early field trials than the late field trials. Outcomes from the one-way ANOVA found no significant main effect of experimental period (*p* = 0.267) when comparing baseline, catch right, and catch left.

Initially when participants performed walking trials in the unpredictable force field, they exhibited large lateral deviations of their COM trajectory in the same direction as the applied force field (right or left depending on the force field direction) (Fig. 1c). As participants practiced walking in the force field, the magnitude of the COM lateral deviation resulting from the applied force field was reduced, becoming more similar to COM trajectories performed during the baseline trials. Specifically, COM signed deviation had a large bias towards the right during the early field right trials, − 0.066 ± 0.032 m^2^ (mean ± SD) (negative values indicate a bias towards the right relative to baseline) and large bias towards the left during the early field left trials 0.055 ± 0.017 m^2^ (Fig. 4). By the late field trials the magnitude of the COM signed deviations had been reduced by 44% (right) and 28% (left). Outcomes from the two-by-two ANOVA, which analyzed adaptation, identified a significant main effect of experimental period (*p* = 0.001) indicating that the magnitude of the COM signed deviation was greater during the early field trials than the late field trials. There was no significant main effect of force field direction (*p* = 0.264), and no significant interaction between experimental period and direction (*p* = 0.478).

### Center-of-mass signed deviation—catch trials

When the force field was unexpectedly removed during catch trials, participants exhibited COM trajectories that were similar to the trajectories performed during baseline trials (Fig. 4). Specifically, the COM signed deviation of catch trials preceded by a right directed force field (catch right) was − 0.002 ± 0.017 m^2^ and catch left trials were − 0.008 ± 0.017 m^2^. Outcomes from the one-way ANOVA found no significant main effect of experimental period (*p* = 0.267) when comparing baseline, catch right, and catch left.

### Center-of-mass signed deviation—trial-by-trial analysis

**Figure 5:**
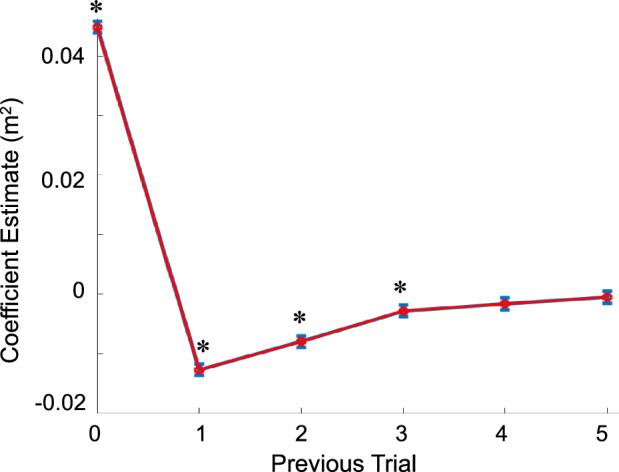
Trial-by-trial analysis Results of a linear regression model between force field direction and COM signed deviation. Variable coefficient estimates are on the y-axis and variable number representing previous trial number are listed on the x-axis. Error is shown as mean $\pm$ SD. Significance from zero (*p* < 0.05) is denoted by (✽). These results suggest that when a given force field trial was implemented this caused error in that direction. On previous trials there was compensation in the opposite direction of the initial error.

Outcomes from our trial-by-trial analysis indicate that COM signed deviation was influenced by force field direction on a given trial and by the force field direction from the three prior trials, as indicated by a statistically significant relationship between force field direction and COM signed deviation (*p* < 0.001, r^2^ = 0.709; Fig. 5). The magnitude of the COM signed deviation on a given trial was positively influenced by the direction of the lateral force field on the current trial and negatively influenced by the force field direction of the three proceeding trials. For example, the effect of a force field directed to the right would be an increase in the COM signed deviation to the right (the same direction as the force field) on the current trial and a bias COM signed deviation to the left (the opposite direction of the force field) on the following three trials, indicating that subjects attempted to learn and correct for past disturbances. The linear regression model resulted in estimated coefficients that were positive (0.045 ± 0.001 m^2^) (mean ± SE) indicating that force field and COM signed deviation were in the same direction and significant for the trial of a given force field direction (*p* < 0.001). Estimated coefficients were negative (indicating that force field and COM signed deviation were in the opposite direction) and significant for the first trial (− 0.013 ± 0.001 m^2^) (*p* < 0.001), second trial (− 0.008 ± 0.001 m^2^) (*p* < 0.001), and third trial (− 0.003 ± 0.001 m^2^) (*p* < 0.004) after a given trial. Estimated coefficients were not significant for the fourth trial (*p* = 0.098) and fifth trial (*p* = 0.605) after a given trial.

### Center-of-mass lateral offset

**Figure 6:**
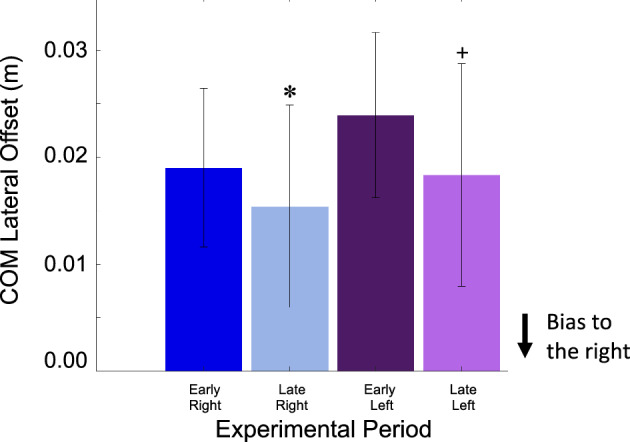
COM Lateral Offset Statistical Results Results of statistical analysis for COM lateral offset. Mean $\pm$ SD for COM lateral offset across experimental period. X-axis labels: ER, EL, LR, and LL represent periods early right, early left, late right, and late left respectively. Significance (*p* < 0.05) from early field right is denoted by (✽) and significance (*p* < 0.05) from early field left is denoted by ( +). Outcomes from the two-by-two ANOVA found a significant main effect of experimental period (*p* = 0.014), indicating that the magnitude of the COM lateral offset was greater during the late field trials than the early field trials. We also found a significant main effect of direction (*p* = 0.032), indicating that force field right trials were more biased towards the right than force field left trials.

As participants walked in the force field their COM lateral offset became increasingly biased towards the right. Specifically, COM lateral offset shifted right from 0.020 ± 0.007 m in early field right to 0.015 ± 0.009 m in late field right (negative values indicate a bias towards the right). COM lateral offset also shifted right from 0.0240 ± 0.008 m (mean ± SD) in early field left and 0.018 ± 0.010 m in late field left (Fig. 6). Outcomes from the two-by-two ANOVA found a significant main effect of experimental period (*p* = 0.014), indicating that the magnitude of the COM lateral offset was greater during the late field trials than the early field trials. We also found a significant main effect of direction (*p* = 0.032), indicating that force field right trials were more biased towards the right than force field left trials. Additionally, we found no significant interaction between experimental period and direction (*p* = 4.02).

### Lateral foot placement

**Figure 7:**
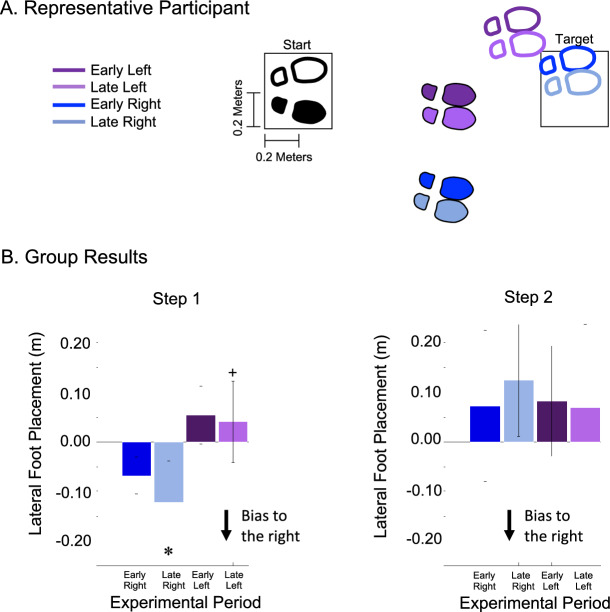
Lateral foot placement (**A**) Representative participant and (**B**) Statistical results (**A**) Foot placement during select trials from analysis periods for a single representative participant. Filled in footprints represent the right foot and outlined footprints represent the left foot. Black and white footprints show feet at start position and colored footprints show step one and step two, respectively. (**B**) Mean $\pm$ SD for lateral foot placement across experimental period. X-axis labels: ER, EL, LR, and LL represent periods early right, early left, late right, and late left respectively. Significance (*p* < 0.05) from early field right is denoted by (✽) and significance (*p* < 0.05) from early field left is denoted by ( +). For step one, outcomes from the two-by-two ANOVA identified a significant main effect of both experimental period (*p* < 0.001), indicating that steps were placed more to the right during late field trials relative to early field trials, and direction (*p* < 0.001), indicating that force field right lateral foot placement was placed farther to the right than force field left, along with no significant interaction between experimental period and direction (*p* = 0.197). For step two, outcomes for the two-by-two ANOVA found no significant main effect of experimental period (*p* = 0.700) or direction (*p* = 0.350) and no significant interaction effect between experimental period and direction (*p* = 0.243).

With experience the lateral foot placement of the first step became increasingly biased towards the right for force fields applied in either direction. Specifically, participants lateral foot place was − 0.068 ± 0.037 m (mean ± SD) (positive values indicate steps that are left of the COM) during early field right which adapted to be placed more to the right during late field right − 0.123 ± 0.084 m (Fig. 7). Similarly, during early field left trials, lateral foot placement was 0.055 ± 0.058 m which adapted to be placed more to the right during late field left trials 0.041 ± 0.082 m. Outcomes from the two-by-two ANOVA identified a significant main effect of both experimental period (*p* < 0.001), indicating that steps were placed more to the right during late field trials relative to early field trials, and direction (*p* < 0.001), indicating that force field right lateral foot placement was placed farther to the right than force field left, along with no significant interaction between experimental period and direction (*p* = 0.197).

With experience lateral foot placement of the second step remained the same as in the early field conditions. Specifically, participants lateral foot place was 0.072 ± 0.153 m (mean ± SD) (positive values indicate steps that are left of the COM) during early field right which remained the same during late field right 0.124 ± 0.112 m (Fig. 7). Similarly, during the early field left lateral foot placement was 0.083 ± 0.111 m which also remained the same during the late field left trials 0.070 ± 0.168. Outcomes from the two-by-two ANOVA found no significant main effect of experimental period (*p* = 0.700) or direction (*p* = 0.350) and no significant interaction effect between experimental period and direction (*p* = 0.243).

## Discussion

This study investigated how people plan their COM trajectory during a goal-directed walking task when a laterally-directed force field was randomly pulled them either to their right or left. When participants initially performed the walking task in the force field they exhibited substantial increases in their COM signed deviation in the same direction as the applied field for both the left and right directed force field trials. As participants practiced walking in the force field, the magnitude of the COM signed deviation was reduced becoming more similar to trials performed during the baseline condition. This finding supports our primary hypothesis that people would adapt a control strategy to reduce the COM lateral deviations created by the novel and unpredictable force field.

To effectively walk from one location to another, it is essential that a person be able to respond to external disturbances. An inability to do so would result in movement errors that may require a corrective step or worse may create a fall or collision with an obstacle. A challenge arises when the external environment is unpredictable because the nervous system is unable to plan specific walking patterns that efficiently and reliably offset destabilizing aspects of the environment. In an unpredictable environment the motor control strategy used to control walking should provide resistance to the range of possible disturbances that may be encountered. However, the strategies people adapt to control walking in unpredictable environments is not clear. In the current experiment, we used a cable-driven robot to create a powerful destabilizing force field that was unpredictably directed to either the left or right. We found that with experience participants adapted a control strategy that substantially reduced the magnitude of the lateral COM deviation created when the force field was applied in either direction. We also found that during catch trials, when the force field was unexpectedly removed, COM trajectories were similar to those performed during baseline trials. As should be expected in an unpredictable environment^8,9,14^ , the absence of after-effects during the catch trial indicates that the control strategy adapted was not predictive of a force field applied in a specific direction. Our findings are consistent with the adaptation of an impedance control strategy to stabilize lateral displacements of the COM during walking in an unpredictable environment. The adaptation of an impedance control strategy that provided a generalized resistance to external perturbations allowed participants to successfully complete a goal directed walking task that had both temporal and spatial requirements in a consistent and reliable manner.

The results of our trial-by-trial analysis indicate that participants were also using a predictive control strategy. Our linear regression model indicates that the direction of the force field applied on a given trial resulted in changes in the COM signed deviation in the opposite direction of the applied force field on the following trial (Fig. 5). Furthermore, COM signed deviation for a given trial was the result of the current trial and three previous trials. Therefore, on a trial-by-trial level, our model indicates that participants were using a predictive strategy to adapt COM trajectory in response to recent experience. Thus, in this short time scale experiment participants were persistent in finding predictable aspects of the environment. This could be because it is likely that we are rarely in a completely random environment.

The presence of two competing strategies is complementary when you look at the scope of each individual analysis. The catch trial analysis shows the cumulative result of all the preceding trials and their effective outcome. Although the trial-by-trial analysis suggests that the effect of any given trial will persist for three trials, in the presence of randomized trials right and left, the effect of each individual trial can be counteracted if the surrounding trials are in the opposite direction. While the presence of two strategies—impedance and predictive—may seem contradictory to learning, when learning to control walking in a novel environment, the presence of these two competing strategies may be valuable. In a novel environment the nervous system will not know if the destabilizing components of the environment are predictable or unpredictable. The presence of an impedance control strategy can produce stable walking trajectories during the initial exposure when it is still unknown if the environment is predictable or unpredictable. At the same time, the presence of a predictive control strategy can allow the nervous system the flexibility to try and identify predictable aspects of the environment. If the environment is indeed learnable, a predictive control strategy is valuable for creating movements because precise motor commands can be generated that directly counter the predicted force field^5–7^ and allow for both stable and energetically efficient movements^50^. Should the novel environment turn out to be unpredictable, as was the case in the current experiment, we would anticipate that over time that the predictive compensatory response to a given force field direction should be gradually diminished^24,25^. As the title of this paper suggests, we define this combination of strategies as “optimism” because the nervous system is continuing to search for predictable aspects of the environment. If predictable aspects of the environment are found, this could lead to better performance, even amid the presence of uncertainty. This is optimism because experience would suggest that this would be unsuccessful in an unpredictable environment.

Participants used two distinct mechanisms to create a bilateral whole-body resistance to the unpredictable force field. First, participants adapted their COM lateral offset to become more biased to the right (Fig. 6). The COM lateral offset is an anticipatory postural adjustment that during a typical gait initiation is used to shift the COM location laterally to unweight the stepping limb^41^. In our prior study^38^ we found that participants adapted the magnitude of their COM lateral offset in the opposite direction of a predictable and consistent laterally-directed force field. The effect of this action preemptively positioned the COM location to counter the effects of the force field once the forward walking trajectory begins. In the current experiment, increasing the COM lateral offset to the right will position the COM location to resist force fields applied to the left. This was a unilateral strategy that increased resistance to force fields applied to the left but placed the COM in a poor location to counteract force fields applied to the right. It is important to note that in the results, the force field right trials significantly deviated towards the right compared to the force field left trials. This is because we calculated lateral offset as the difference in lateral position at the start of the task and at right foot toe-off. We selected a forward velocity of 0.26 m/s to trigger the force field onset based on pilot data that found on average this threshold was reached immediately after right toe-off. However, due to intra- and inter-subject variations, on some trials the force field began just prior to toe-off which likely resulted in the differences between right and left force field trials. We don’t believe that this significantly influenced our overall findings. Second, participants adapted the lateral foot placement location of their first step (always performed with the right leg) (Fig. 7). With experience in the unpredictable force field, the location of the first step was placed farther to the right. The placement of the first step farther to the right occurred for force fields applied to either the right or the left. A lateral adjustment of foot placement farther to the right will increase resistance to force fields applied to the right but will place the body in a poor position to resist forces applied to the left. Thus, the adaptation of a wider step to the right was a unilateral strategy increasing resistance in only one direction.

It is important to note that step one was primarily analyzed because we were most interested in strategies that contributed to predictive control. Due to the timing, step two has significantly more feedback information available and therefore likely relies on a combination of both predictive and reactive control strategies. Furthermore, another study has found that people primarily use stepping strategies when perturbed at walking speeds of 0.8 m/s or higher as opposed to a stance response that includes a combination of ankle and hip strategies when walking at slower speeds^50^. At faster speeds the change to a stepping strategy is necessary to generate a sufficient lateral impulse. This is because the associated shortening of the stance phase duration will limit the mediolateral impulse that can be created by the ankle and hip strategies. These previous observations are consistent with our study which found that people primarily use a stepping strategy when responding to the lateral force fields during the rapid stepping task.”

By themselves neither strategy should be effective for increasing bilateral resistance to the unpredictable force field. In fact, when considered independently, it would be anticipated that with experience in the unpredictable force field that a participant would select a foot placement location and COM lateral offset that are optimized to maximize resistance in both directions, adapting a strategy that is the best response to the average direction of the unpredictable force field, such as choosing a COM lateral offset that is neither bias to the left or right. However, this was not how participants adapted these two control mechanisms. We observed that the two mechanisms were adapted in a synergistic manner, foot placement adaptations were used to increase resistance to force fields directed to the right and COM lateral offset adaptations were used to increase resistance to force fields directed to the left.

Previous studies have looked at differences in control strategy between inward and outward perturbations^51–53^. In the current study, participants stepped with their right foot first and forces were applied to either the right or left. When forces were applied to the right, this would create an outward perturbation on step one. When forces were applied to the left, this would create an inward perturbation on step one. These prior studies found that outward perturbations were more complex and involved various strategies including hip/ankle strategies as well as stepping strategies, while inward perturbations were less complex and primarily involved a stepping strategy. In this study, we found that COM signed deviation was reduced by 28% for the left force field and 44% for the right force field. The greater reduction in COM signed deviation for the outward perturbation created by the right force field could be due to the availability of a greater number of possible strategies that could be used to adapt in the force field when compared to the left force that created an inward perturbation.

## Conclusions

We found that participants adapted a control strategy that was effective for reducing the magnitude of lateral COM deviations in response to unpredictable force fields applied in either the left or right direction. Impedance control was an important component of the strategy adopted by all subjects. In addition to impedance control, we found evidence for predictive control, even though effective predictions were not possible for the random disturbances that the participants’ experienced. The presence of these competing control strategies created movement errors on certain trials but may have long-term benefits by allowing the nervous system to identify the best overall control strategy to use in a novel environment. Additionally, participants adapted two distinct unilateral strategies that collectively created a bilateral resistance to the unpredictable force field. These strategies included an anticipatory postural adjustment to resist against forces applied to the left, and a wider first step to resist against forces applied to the right.

## Data Availability

The data generated and analyzed during this current study is available in Open Science Framework, https://osf.io/q8bf9/.
